# A CNN-LSTM model for six human ankle movements classification on different loads

**DOI:** 10.3389/fnhum.2023.1101938

**Published:** 2023-03-08

**Authors:** Min Li, Jiale Wang, Shiqi Yang, Jun Xie, Guanghua Xu, Shan Luo

**Affiliations:** ^1^Department of Mechanical Engineering, Xi’an Jiaotong University, Xi’an, China; ^2^Department of Engineering, King’s College London, London, United Kingdom

**Keywords:** SEMG signal, ankle movement classification, load variation, CNN, LSTM

## Abstract

This study aims to address three problems in current studies in decoding the ankle movement intention for robot-assisted bilateral rehabilitation using surface electromyogram (sEMG) signals: (1) only up to four ankle movements could be identified while six ankle movements should be classified to provide better training; (2) feeding the raw sEMG signals directly into the neural network leads to high computational cost; and (3) load variation has large influence on classification accuracy. To achieve this, a convolutional neural network (CNN)—long short-term memory (LSTM) model, a time-domain feature selection method of the sEMG, and a two-step method are proposed. For the first time, the Boruta algorithm is used to select time-domain features of sEMG. The selected features, rather than raw sEMG signals are fed into the CNN-LSTM model. Hence, the number of model’s parameters is reduced from 331,938 to 155,042, by half. Experiments are conducted to validate the proposed method. The results show that our method could classify six ankle movements with relatively good accuracy (95.73%). The accuracy of CNN-LSTM, CNN, and LSTM models with sEMG features as input are all higher than that of corresponding models with raw sEMG as input. The overall accuracy is improved from 73.23% to 93.50% using our two-step method for identifying the ankle movements with different loads. Our proposed CNN-LSTM model have the highest accuracy for ankle movements classification compared with CNN, LSTM, and Support Vector Machine (SVM).

## 1. Introduction

In the World Health Organization report on aging and health, it is noted that the proportion of older people in the world will exceed 25% by 2029 (World Health Organization, 2015). Stroke is one of the most common diseases among older people, and motor impairments of limbs, such as foot drop and spastic equinovarus foot deformity are one of the most common outcomes after stroke (Leardini et al., 2019). The ankle joint has many important functions including assisting people walking, supporting the body’s weight, maintaining balance, and changing posture (from sitting to standing or from lying to sitting; Jung, 2016; Zhang et al., 2022). Normal functioning in people’s daily lives needs a normal ankle joint; mobility deficits on the ankle joint can lead to difficulties in walking and other activities of daily life (Dettwyler et al., 2004; Jamwal et al., 2014). The ability to control the body is inversely proportional to the distance between the brains and limbs, implying that the greater the distance, the lower the ability (Gwin and Ferris, 2012). Therefore, the recovery of the motor function of ankles is more difficult than that of other joints with a similar disability (Zeng et al., 2017). For stroke survivors, ankle joints can gradually stiffen without adequate exercises and eventually develop foot drop (McCrimmon et al., 2015). Ankle muscle stretching and joint rotation training may be beneficial for early-stage stroke survivors who are not yet capable of performing multi-joint training exercises.

Bilateral training, which involves using both limbs/sides of the body simultaneously, is a commonly used stroke rehabilitation method (Cauraugh et al., 2010). Most stroke patients have unilateral motor dysfunction or hemiplegia (Yue et al., 2017). One side of the patient’s ankle is intact, which is called the unaffected limb. The other side of the ankle has different degrees of motor dysfunction, which is called the affected limb. Bilateral training applies interneural coordination assumptions to activate the motor synergy between limbs. In other words, when performing symmetrical movements, activating the primary motor cortex and supplementary motor areas of the unaffected limb increases the likelihood of spontaneous muscle contractions (i.e., motor synergy) in the affected limb (Stewart et al., 2006). Bilateral training can effectively activate the brain areas of the human body, such as auxiliary motor area and sensory motor cortex (Waller et al., 2006; Cauraugh et al., 2010). Evidence shows that the simultaneous movement of both limbs can help the neuromuscular system regain certain stability and improve the use of affected limbs (Waller et al., 2006). Therefore, it has a better rehabilitation effect (Oujamaa et al., 2009; Zhu et al., 2021).

Many studies have shown that robot-assisted training can restore the motor functions of patients (Jamwal et al., 2014; Zhang et al., 2017; Akbari et al., 2021). Passive and active robot-assisted training has been used for stroke rehabilitation (Ang et al., 2014). During active robot-assisted rehabilitation, patients are motivated. Therefore, active robot-assisted training can accelerate the recovery of the central nervous system compared with passive robot-assisted training (Stewart et al., 2006; Miao et al., 2018). Robot-assisted bilateral training combines the concept of bilateral training with active robot-assisted training (Ueki et al., 2012; Leonardis et al., 2015). In robot-assisted bilateral training, the robot-assisted movements of the affected limb are often controlled by sensing the voluntary movements of the unaffected limb (Ueki et al., 2012; Leonardis et al., 2015). Robot-assisted bilateral training has shown promising results as an effective limb rehabilitation strategy for stroke patients (Leonardis et al., 2015). To achieve this type of robot-assisted bilateral training, decoding the movement intention of the unaffected limb is necessary to control the robot placed on the affected side. Surface electromyography (sEMG) signals are widely used to decode human movement intentions during rehabilitation training (Varol et al., 2010; Joshi et al., 2013; Liu et al., 2019; Gautam et al., 2020; Zhang et al., 2021, 2022) For lower-limb rehabilitation training, the current literature mainly focuses on gait recognition rather than ankle movement classification (Varol et al., 2010; Joshi et al., 2013; Liu et al., 2019; Gautam et al., 2020; Zhang et al., 2021, 2022). However, gait recognition is applicable to individuals with sufficient walking ability, whereas platform-based ankle rehabilitation robots are more suitable for early stroke patients. Therefore, classifying the ankle movements through sEMG signals is necessary to achieve bilateral training for individuals with weak motor ability who can only use platform-based ankle rehabilitation rather than gait rehabilitation robots (Zhang, 2016; Zeng et al., 2017).

Only a few studies have classified ankle movements from sEMG signals (Chen et al., 2019; Cheng et al., 2020; Hooda and Kumar, 2021). Among these studies, Chen et al. (2019) classified two ankle movements using their method, including inversion (IV) and eversion (EV). Studies have shown that the plantarflexion (PF) and dorsiflexion (DF) of the ankle joint play an essential role in human walking (Huang et al., 2015). Therefore, Hooda and Kumar (2021), as well as Cheng et al. (2020), classified four ankle movements: PF, DF, EV, and IV. Apart from PF, DF, EV, and IV, the ankle joint can also perform internal rotation (IR) and external rotation (ER) movements (Meng et al., 2014, Brockett and Chapman, 2016). According to the research by Meng et al. (2014); there are two peaks of IR torque and one peak of external rotation torque in the ankle joint during normal walking. In other words, IR and ER movements occur in normal human walking. Moreover, during a DF movement, an IR movement also occurs while during a PF movement, an ER movement also occurs (Brockett and Chapman, 2016). Therefore, all six ankle movements should be classified to provide better ankle rehabilitation training. To the best of our knowledge, no studies have reported using sEMG signals to classify all these six ankle movements.

The introduction of deep learning methods in motion intention recognition using sEMG signals has led to good classification results (Huang et al., 2019; Gautam et al., 2020; Lai et al., 2021; Nguyen-Trong et al., 2021; Tripathi et al., 2022; Yang et al., 2022). Regarding ankle movement classification, Chen et al. (2019) used a cerebellar model neural network (CMNN) to classify two ankle movements (IV and EV). They achieved a high classification accuracy of 96.90%. Cheng et al. (2020) proposed a convolutional neural network (CNN)—long short-term memory (LSTM) hybrid model to classify four ankle movements (DF, PF, IV, and EV). They tested this model on three healthy subjects with an average accuracy of 97.55%, which is a great improvement over traditional machine learning classifiers. However, many previous studies mainly focused on improving the accuracy of classification rather than the real-time performance of online classification (Cheng et al., 2020; Gautam et al., 2020; Lai et al., 2021; Nguyen-Trong et al., 2021; Tripathi et al., 2022; Yang et al., 2022), which is extremely important for bilateral training. The original signals are often directly fed into the deep learning models (Cheng et al., 2020; Gautam et al., 2020; Lai et al., 2021; Nguyen-Trong et al., 2021; Tripathi et al., 2022; Yang et al., 2022); therefore, even a slightly large amount of data can seriously affect the real-time performance (Joshi et al., 2013). Moreover, these deep learning models have a large number of parameters when raw sEMG signals are used, which leads to high computational costs, making their application difficult for occasions requiring real-time performance (Xia et al., 2020). For example, Atzori et al. (2016) proposed a CNN to classify hand movements by sEMG. They fed the preprocessed sEMG directly into CNN. The average time required to train CNN was 1 h and 42 min, and the average time required to test the network was 21.5 s using an Nvidia Titan-x GPU (Atzori et al., 2016). Liu et al. (2019) used CNN to continuously estimate the knee joint angle by sEMG. Two neural networks were trained on a system with Intel (R) Core (TM) i7-8700K CPU (Intel, Santa Clara, CA, USA), a 3.70 GHz processor and 31.3 GB of RAM: original data-based CNN and feature-based CNN. The training time for original data-based CNN was 5,400 ± 25 s, and for feature-based CNN was 31.7 ± 3.6 s, which meant that the training time of using features as CNN input was greatly reduced (Liu et al., 2019). Fewer studies have tried to use extracted features instead of original sEMG signals as the input of deep learning models in motion classification. Chen et al. (2019) tried to extract the root mean square (RMS) feature of the EMG signal as the input of CMNN and realized the classification of two movements of the ankle joint. Using RMS had the advantages of high real-time performance and low computational complexity (Chen et al., 2019). Unfortunately, they only considered the feature of RMS, namely, they ignored the information other than RMS in sEMG signals. Moreover, they failed to compare their method with the traditional methods to verify the advantages of their method. To the best of our knowledge, no study has attempted to use feature selection algorithms for selecting appropriate features to form a feature set as the input of deep learning models so as to improve the real-time performance in ankle movement classification.

Moreover, the load variation greatly influences the classification accuracy in motion recognition using sEMG signals (Al-Timemy et al., 2013; Tang et al., 2016). When the same movement is performed with different loads, the features such as RMS of sEMG are different. At this point, if we continue to use the trained classifier under a single load, the accuracy will be much lower. Studies have shown that the load variation may lead to a decrease in the accuracy of sEMG recognition by up to 60% (Al-Timemy et al., 2013) and also an increase in Root Mean Square Error (RMSE) for the joint angle from 7.86° to 20.44° (Tang et al., 2016). Moreover, Tang et al. (2016) found that only putting EMG signals under all loads together into the model could not achieve an ideal performance when decoding elbow movements. To the best of our knowledge, no study has provided solutions to reduce the influence of load variation on the classification accuracy in ankle movement classification.

In summary, three problems exist in decoding the ankle movement intention for robot-assisted bilateral rehabilitation using surface electromyogram (sEMG) signals: only up to four ankle movements can be identified, whereas six ankle movements should be classified to provide better training; feeding the raw sEMG signals directly into the neural network leads to high computational cost; and load variation has a great influence on classification accuracy. To solve the aforementioned problems, a time-domain feature selection method of the sEMG, a CNN-LSTM model, and a two-step method are proposed in this study to decode the movement intention of the ankle joint for bilateral ankle rehabilitation training. First, the Boruta algorithm is used to select relevant features, and the corresponding feature matrix is calculated. Then, the feature matrix instead of the original signal is fed into the deep learning model, which greatly reduces the computational cost. In addition, a two-step method is adopted. We first identify the load on the ankle joint and then identify the movement, which greatly reduces the impact of the load variation on accuracy. Compared with previous studies, our major contributions are as follows: (a) the classified ankle movements are expanded from four to six; and (b) the influence of the load variation on movement recognition is reduced.

The structure of the remainder of this article is as follows. Section “1 Introduction” introduces the methodology of our proposed methods. Section “2 Materials and methods” describes the validation experiments. Section “3 Experiment and results” discusses the experiment results, and Section “4 Discussion” draws a conclusion.

## 2. Materials and methods

**Figure 1:**
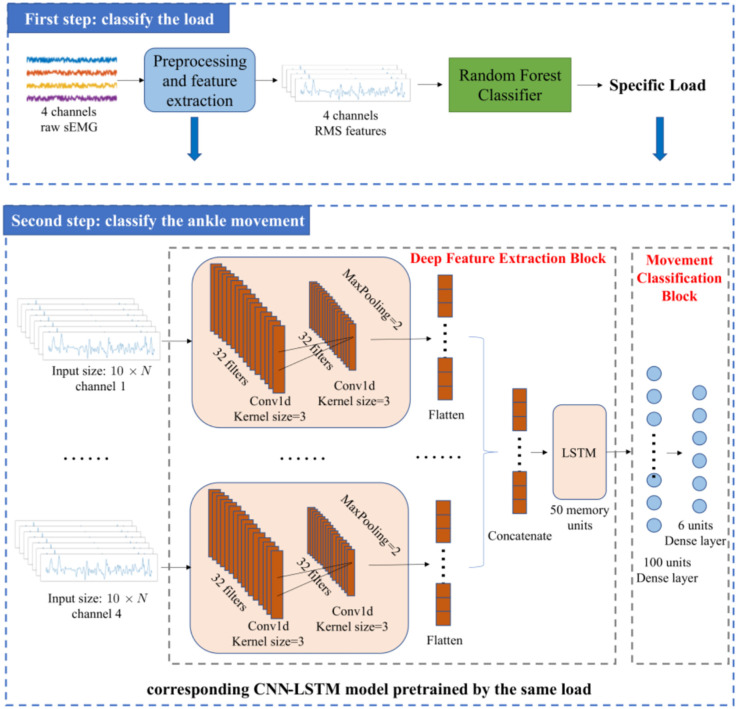
Framework of the ankle movement classification method.

The framework of the ankle movement classification method proposed in this article is shown in Figure 1. First, four channels of raw sEMG signals were collected. Then, the raw sEMG signals were preprocessed through notch filtering, bandpass filtering, and normalization (Rajapriya et al., 2021). Next, a two-step method was adopted to improve the accuracy of ankle movement classification with different loads. In the first step, the RMS of sEMG was calculated and classified using a Random Forest classifier to obtain the specific load including low, medium, and high. In the second step, seven most relevant time-domain features were selected using the Boruta algorithm (Kursa and Rudnicki, 2010) and fed into the corresponding CNN-LSTM model to classify the ankle movement with the aim of reducing the computation cost and improve the accuracy. The detailed process is described in the following paragraphs.

### 2.1. Data acquisition

**Figure 2:**
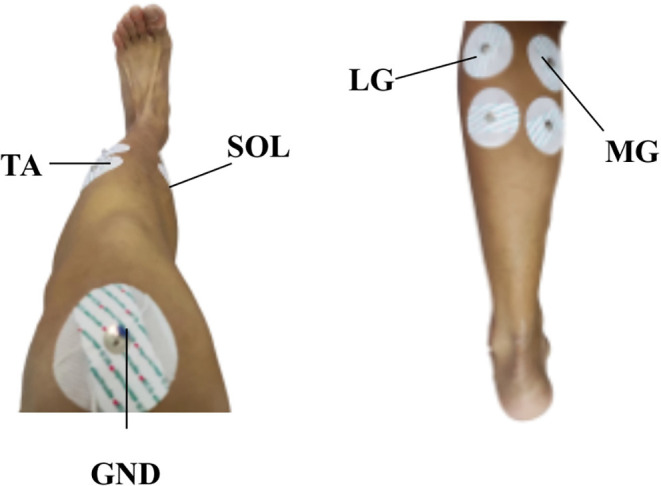
EMG electrodes positions.

The target movements of the ankle joint in this study include: PF, DF, IV, EV, IR, and ER. Four muscles, including tibialis anterior muscle (TA), lateral gastrocnemius muscle (LG), medial gastrocnemius muscle (MG), and soleus muscle (SOL) play an important role in these target ankle joint movements. Therefore, these four muscles were selected to collect sEMG signals (Al-Quraishi et al., 2015). The bipolar sEMG electrodes on the left shank were positioned according to the Surface EMG for Non-Invasive Muscle Evaluation (SENIAM, seniam.org) criteria. As shown in Figure 2, the electrodes of 1–4 were attached to the TA, LG, MG, and SOL of each subject, respectively.

**Figure 3:**
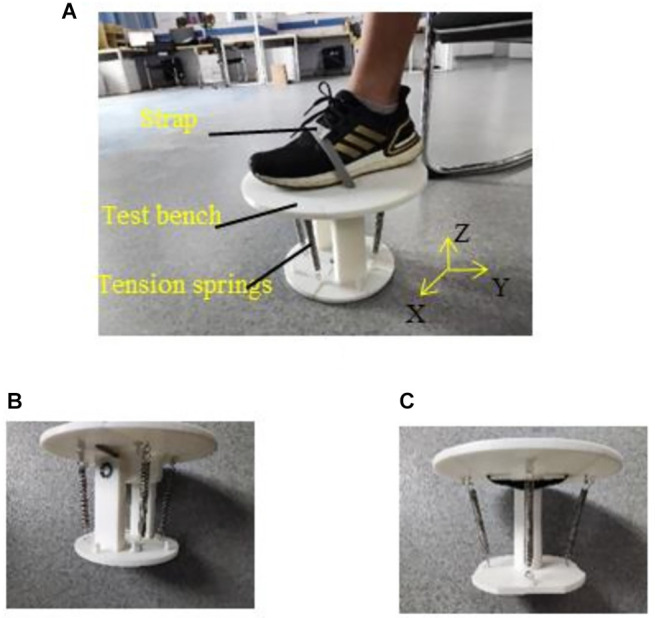
Test benches. **(A)** Experimental setup. **(B)** Test bench of DF, PF, IV, and EV, the moving platform could rotate around the *x*-axis and *y*-axis. **(C)** Test bench of IR and ER, the moving platform could rotate around the *z*-axis.

The physiological space of the ankle joint can be divided into the frontal axis, the sagittal axis, and the vertical axis to facilitate the analysis of the ankle joint (Dettwyler et al., 2004). PF and DF movements rotate around the frontal axis; IV and EV movements rotate around the sagittal axis; and IR and ER movements rotate around the vertical axis. Therefore, two test benches were designed to simulate the load variation on the ankle, as shown in Figure 3. The test bench was composed of a static platform and a moving platform, which could rotate relative to the static platform. The test bench shown in Figure 3B could rotate around the frontal and sagittal axes, and therefore PF/DF and IV/EV movements could be simulated with this test bench. The test bench shown in Figure 3C could rotate around the vertical axis, and therefore IR/ER movements could be simulated with it. Four tension springs with the same stiffness were used to connect the static and the moving platforms. They were placed at the same distance and angles as their neighbors. When the moving platform rotated relative to the static platform, the tension of the spring needed to be overcome. Three kinds of tension springs with different stiffness values (0.04 N/mm, 0.15 N/mm, and 0.41 N/mm) were used to simulate the low, medium, and high loads on the ankle joint, respectively. Regarding the tension spring stiffness, the higher load was roughly three times of the lower load. The torque generated by the tension spring with the largest stiffness (0.41 N/mm) when performing PF, DF, IV, EV, IR, and ER was 3.05, 3.39, 3.39, 3.33, 2.38, and 3.44 Nm, respectively. The torque of normal people when performing PF, DF, IV, EV, and IR/ER, tested in a seated posture task, is 6.11, 11.59, 3.62, 4.18, and 4–8 Nm, respectively (Lu, 2016; Chen, 2018). For hemiplegics, the torques performing these movements will be reduced to about 20.40%–30.60% (Kim and Eng, 2003). Therefore, our device is suitable for simulating the ankle movements of patients with hemiplegia.

### 2.2. Data pre-processing

In general, the sEMG signals need to be segmented into smaller sections before signal processing. A sliding window is applied. The control delay should be less than 300 ms to realize an effective real-time control for the rehabilitation robot (Hudgins et al., 1993). Moreover, some studies suggested that the sliding window using 100–300 ms window length with 20%–60% overlapping is suitable for real-time control for the rehabilitation robot (Rajapriya et al., 2021; Teng et al., 2021). Therefore, the window length was set to be 210 ms and the sliding length was 120 ms.

The power-line interference was removed by applying a 50 Hz notch filter on the raw sEMG signals (Rajapriya et al., 2021). The frequency of sEMG is concentrated at 20−450 Hz, and the voltage amplitude of sEMG is low and varies weakly, making it easily submerged by noise signals (Al-Quraishi et al., 2015). To avoid this, a 4th Butterworth bandpass filter with a cut-off frequency of 20−450 Hz was used to filter and reduce the noise. Finally, in order, the filtered sEMG was normalized into zero mean and unit variance to accelerate the convergence of neural network (Sola and Sevilla, 1997).

### 2.3. Feature extraction

Previous studies explored 37 time-domain and frequency-domain features (Phinyomark et al., 2012). They evaluated these features through the scatter plot of features, statistical analysis, and classifier methods. They found that all frequency-domain features were calculated based on the statistical parameters of EMG power spectral density and were not suitable for EMG recognition systems. The time-domain features have a higher correlation with movement and are considered to have better performance than frequency-domain and time-frequency-domain features (Phinyomark et al., 2012; Al-Quraishi et al., 2015). Therefore, time-domain features were selected to form the feature vector in this study. Time-domain features commonly include the following: (a) RMS; (b) Mean Absolute Value (MAV); (c) Waveform Length (WL); (d) Zero Crossing points (ZC); (e) number of Slope Sign Change (SSC); (f) Variance (VAR); (g) Logarithmic Detection value (LogD); and (h) Willison Amplitude (WA). It is worth mentioning that each of these features used in our study is a one-dimensional feature, which implying that it is calculated as a one-dimensional rather than a multi-dimensional vector. For the given EMG sampled signal *x_i_* of length *M*, the features were calculated using the equations shown in Table 1 (Hudgins et al., 1993; Reaz et al., 2006; Tkach et al., 2010; Kim et al., 2011; Phinyomark et al., 2012, 2013; Al-Timemy et al., 2016). There, the value of the threshold is chosen as 50 μv to avoid background noise (Phinyomark et al., 2012).

**Table 1:** Time-domain features with a mathematical formulation.

**Name**	**Mathematical formulation**
Root Mean Square (RMS; Al-Timemy et al., 2016)	$RMS=\sqrt{\frac{1}{M}\sum_{i=1}^{M}x_{i}^{2}}$
Mean Absolute Value (MAV; Kim et al., 2011)	$MAV=\frac{1}{M}\sum_{i=1}^{M}\left|x_{i}\right|$
Waveform Length (WL; Phinyomark et al., 2013)	$WL=\sum_{i=1}^{M=1}\left|x_{i+1}-x_{i}\right|$
Zero Crossing Points (ZC; Hudgins et al., 1993)	$ZC=\sum_{i=1}^{M=1}\left[\mathrm{sgn}\left(x_{i}\times x_{i+1}\right)\cap \left|{}_{i}-x_{i+1}\right|\geq threshold\right]$ $\mathrm{sgn}\left(x\right)=\left\{\begin{array}{c} 1,\;if\;x\geq threshold\\ 0,\;othrewise \end{array}\right.$
Number of Slope Sign Shange (SSC; Hudgins et al., 1993)	$SSC=\sum_{i=2}^{M-1}\left[f\left[\left(x_{i}-x_{i-1}\right)\times \left(x_{i}-x_{i+1}\right)\right]\right]$ $f\left(i\right)=\left\{\begin{array}{c} 1,\;if\;x\geq threshold\\ 0,\;othrewise \end{array}\right.$
Variance (VAR; Reaz et al., 2006)	$VAR=\frac{1}{M-1}{\sum_{i=1}^{M}\left(x_{i}-\frac{1}{M}\sum_{i=1}^{M}x_{i}\right)}^{2}$
Logarithmic Detection Value (LogD; Tkach et al., 2010)	$Log\;D=e^{\frac{1}{M}\sum_{i=1}^{M}\log\left|x_{i}\right|}$
Willison Amplitude (WA; Phinyomark et al., 2012)	$WA=\sum_{i=1}^{M-1}\left[f\left(\left|x_{n}-x_{n+1}\right|\right)\right]$ $f\left(i\right)=\left\{\begin{array}{c} 1,\;if\;x\geq threshold\\ 0,\;otherwise \end{array}\right.$

**Figure 4:**
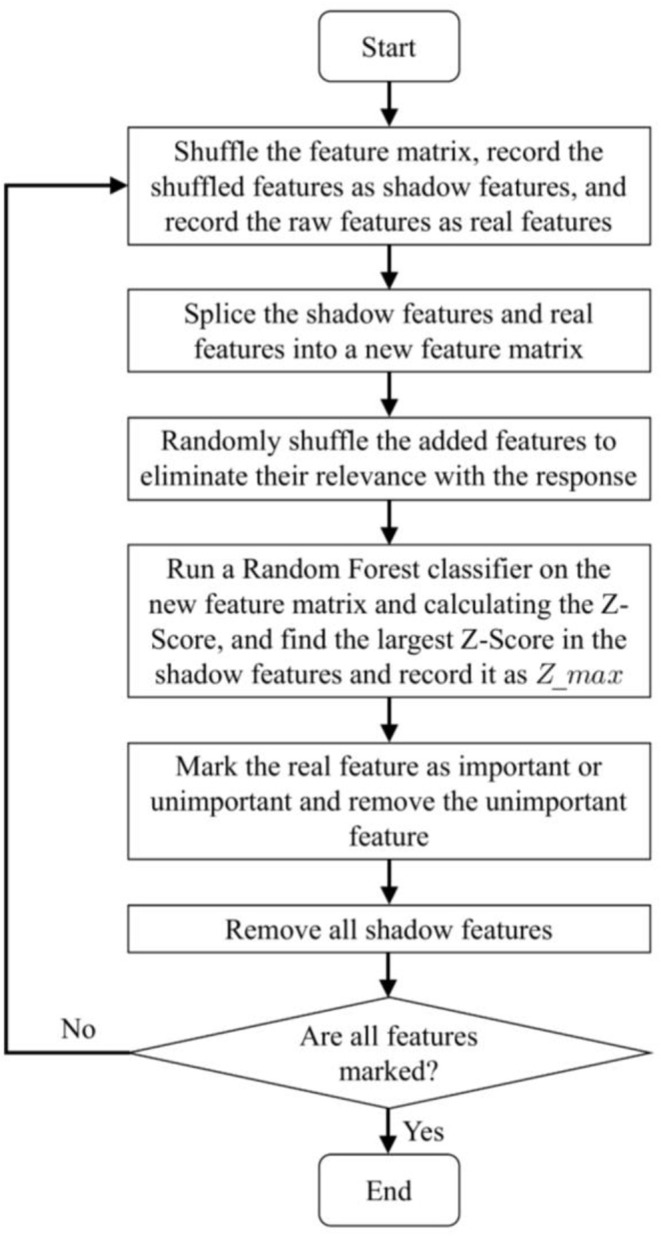
Boruta algorithm procedures.

As mentioned in the introduction, a two-step method is adopted in our study to reduce the impact of load variation on classification accuracy. RMS is usually used for force estimation, which can reflect the level of muscle contraction (Harrach et al., 2017). Therefore, in the first step, RMS was selected as the input of the Random Forest classifier to identify the load levels. The Random Forest classifier was selected to recognize the load level in this article because of its remarkable robustness and good resistance to over fitting (Zhou et al., 2019). In this study, the loads were classified into three load levels (low, medium, and high). The details of the load levels are provided in Section “2.1 Data acquisition”. In the second step, the ankle movements were classified using the feature vector constructed by multiple features as the input of the CNN-LSTM model. Selecting all the aforementioned features will cause a large dimension of the feature vector and a high computation cost. As a result, the training process of the neural network may take a longer time. Meanwhile, redundant features may reduce the accuracy and the performance of classification (Chen et al., 2020). Therefore, selecting several most relevant features from these commonly used time-domain features is necessary. Boruta algorithm is a feature selection algorithm, which is a package based on the Random Forest classification algorithm (Kursa and Rudnicki, 2010; Ahmadizadeh et al., 2019). It provides an unbiased and stable selection of important and unimportant features (Kursa and Rudnicki, 2010). Its main steps are shown in Figure 4.

1. Shuffling the feature matrix, and splicing the shuffled features recorded as shadow features and the raw features recorded as real features into a new feature matrix (Kursa and Rudnicki, 2010).
2. Randomly shuffling the added features to eliminate their relevance with the response.
3. Running a Random Forest classifier on the new feature matrix and calculating the *Z-Score*. The *Z-Score* can represent the relative position of feature importance in the distribution. It is equal to the average feature importance minus the feature importance and then divided by the standard deviation of the feature importance. There, the feature importance is returned by the Random Forest algorithm.
4. Finding the largest *Z-Score* in the shadow features and record it as *Z*_max_.
5. Marking the real feature as important if the *Z-Score* is significantly greater than the *Z*_max_, and marking the real feature as unimportant and permanently eliminating it from the feature set if the *Z-Score* is significantly less than *Z*_max_.
6. Removing all shadow features.
7. Repeating the procedure until all features are marked as important or unimportant.

**Figure 5:**
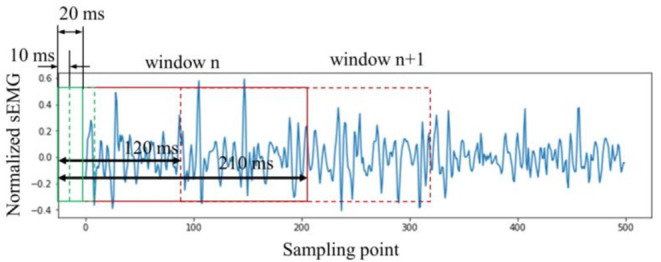
Window splitting method.

In this study, the Boruta algorithm was used to select the time-domain features of the sEMG signals. As mentioned earlier, the window length was 210 ms. Unlike using raw sEMG as input directly, each large window (210 ms) was sectioned into 20 small windows with a window length of 20 ms and a sliding length of 10 ms. The features were then calculated in each 20 ms window. In other words, the large window (210 ms) could be sectioned into 20 small windows (20 ms). The specific window segmentation method is shown in Figure 5. Finally, for each channel, a feature matrix with the size of 20 × *N* was then obtained. *N* represents the number of features selected using the Boruta algorithm.

### 2.4. Network architecture

Figure 1 shows the network architecture of the movement classifier proposed in this article. The features learned by the neural network were referred as deep learning features in this study to avoid confusion with the hand-crafted time-domain features mentioned earlier. The network structure adopted the combination of CNN and LSTM. CNN can extract the deep learning features of sEMG signals (Bao et al., 2021; Zhu et al., 2021), but it cannot extract the temporal correlations. Since sEMG signals have a certain coherence in the time domain, a layer of LSTM, which is a recurrent neural network (RNN) that can extract the deep learning features of the time sequence of sEMG (Xia et al., 2020), was added to the network. In this way, the temporal-spatial correlations of sEMG can be exploited.

The inputs of the network were the feature vectors converted from the feature matrices of the four channels. After the preprocessing, a feature matrix with the size of 20 × *N* was obtained for each channel, but the input of CNN-LSTM is 10 × *N*. This was because the time step of LSTM was set to be 2 and half of the 20 × *N* feature matrix was used as the input in each step. The output of the network is the movement category of the ankle joint, implying that the network is a six-class classifier.

In general, the network comprises a deep feature extraction block and a movement classification block. The deep feature extraction block is composed of a two-layer convolution layer, a maximum pooling layer, and an LSTM layer. Each convolution layer follows a Relu activation function. Then, the outputs of the four channels are concatenated. The concatenated deep learning features are sent to the LSTM layer, followed by the dropout layer. In the next stage, the output of the LSTM layer is fed into the dense layer, followed by a dropout layer and another dense layer that outputs the probabilities of six ankle movements. The movement can be predicted by:


(1) $$\overset{\wedge }{y}=\left\{\begin{array}{c} \arg\max p\left(y\left|X;\emptyset_{l}\right.\right)\;if\;in\;low\;land\\ \arg\max p\left(y\left|X;\emptyset_{m}\right.\right)\;if\;in\;mid\;land\\ \arg\max p\left(y\left|X;\emptyset_{n}\right.\right)\;if\;in\;high\;land \end{array}\right.$$

where *Ø_l_, Ø_m_*, and *Ø_h_* are the parameters of our CNN-LSTM model in specific load, *X* represents the feature matrix of model input, and *y*∈*Y*= {0, 1, 2, 3, 4, 5} denotes the corresponding label set.

Not only does the architecture of the neural network affect its performance, but the selection of hyperparameters is also important (Joshi et al., 2013). For the network architecture proposed in this article, the hyperparameters that needed to be considered included the following: (a) the number of layers of the network, (b) filters size, (c) kernel size, (d) pooling size, (e) stride rate, and (f) time step. At the same time, the training parameters that we needed to adjust included the following: (a) number of epochs, (b) learning rate, (c) activation function, (d) loss function, (e) batch size, and (f) dropout. The hyperparameters of our model were mainly determined with reference to previous studies and then manually tuned *via* experience (Gautam et al., 2020). In the deep feature extraction block, the first convolution layer used 32 filters of size 3 and a stride rate of 1. The second convolution layer also used 32 filters of size 3 and a stride rate of 1. The pool size of the max-pooling layer was 2. A size of 50 memory units was used for the LSTM layer. Meanwhile, the time step of the LSTM layer was set to be 2. The number of neurons in the first dense layer was 100. The number of neurons in the second dense layer is the types of ankle movements, which was set as 6 in this article. The probability of dropout was set to 60%. The purpose of setting the dropout layer was to prevent overfitting. Adam optimizer was used with a learning rate of 0.001. The purpose of this network is to classify ankle movements; therefore, we use a cross entropy loss function *L*:


(2) $$L=\frac{1}{N}\sum_{i}L_{i}=-\frac{1}{N}\sum_{i}\sum_{c=1}^{M}y_{ic}\log\left(p_{ic}\right)$$

where *N* is the sample size in the current batch, *M* is the number of categories, *y_ic_* indicates whether the category is the same as the category of sample *i* (the same is 1 and the different is 0), and *p_ic_* indicates the prediction probability that the observed sample *i* belongs to category c.

According to the relevant literature (Gautam et al., 2020; Bao et al., 2021), the batch size was set to be 256 and the number of epochs was set to be 100 in the training process. The specific parameters of the network model and the size of the deep learning feature vector of each layer are shown in Table 2. The model training and testing were performed on a PC that had AMD Ryzen 7 5800H CPU with 3.20 GHz, 16 GB RAM, and an NVDIA GeForce RTX 3050 Ti graphics card with 4 GB memory.

**Table 2:** Our model parameters details.

**Block**		**Our Model (With sEMG features as input)**		**Our Model (With raw sEMG as input)**	
		**Output Shape**	**Parameters**	**Output Shape**	**Parameters**
Deep Feature Extraction Block	Conv1D_1	(10, 32)	704	(25, 32)	128
	Conv1D_2	(10, 32)	704	(25, 32)	128
	Conv1D_3	(10, 32)	704	(25, 32)	128
	Conv1D_4	(10, 32)	704	(25, 32)	128
	Conv1D_5	(10, 32)	2,080	(25, 32)	2,080
	Conv1D_6	(10, 32)	2,080	(25, 32)	2,080
	Conv1D_7	(10, 32)	2,080	(25, 32)	2,080
	Conv1D_8	(10, 32)	2,080	(25, 32)	2,080
	Maxpooling1D_1	(5, 32)	0	(12, 32)	0
	Maxpooling1D_2	(5, 32)	0	(12, 32)	0
	Maxpooling1D_3	(5, 32)	0	(12, 32)	0
	Maxpooling1D_4	(5, 32)	0	(12, 32)	0
	Flatten_1	160	0	384	0
	Flatten_2	160	0	384	0
	Flatten_3	160	0	384	0
	Flatten_4	160	0	384	0
	Concatenate	(640)	0	1536	0
	LSTM	50	138,200	50	317,400
	Dropout_2	50	0	50	0
Movement Classification Block	Dense_1	100	5,100	100	5,100
	Dropout_3	100	0	100	0
	Dense_2	6	606	6	606
Total Parameters			155,042		331,938

## 3 Experiment and results

### 3.1. Experimental protocol

Six male able-bodied subjects (age: 22–25 years old; height: 170–179 cm; weight: 60–75 kg) participated in this study. The subjects were asked to sit in a chair with their shanks dropping naturally and their whole body relaxed. This study was reviewed and approved by the Institutional Review Board of Xi’an Jiaotong University (Approval No. 2019-584).

**Figure 6:**
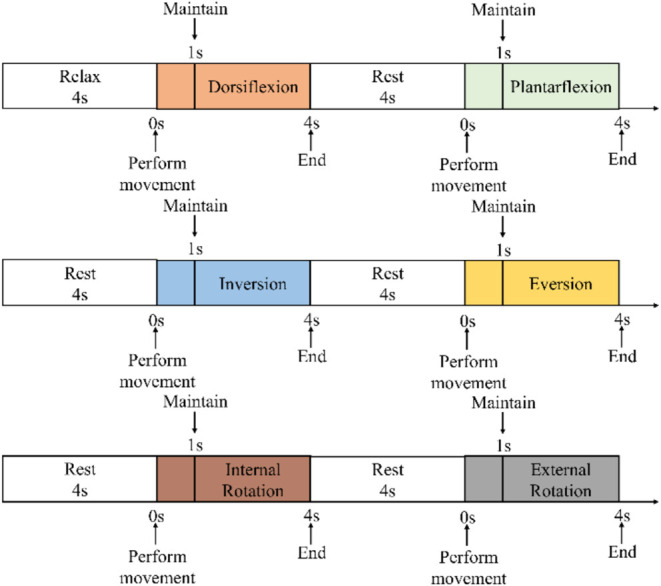
Experimental procedure.

**Figure 7:**
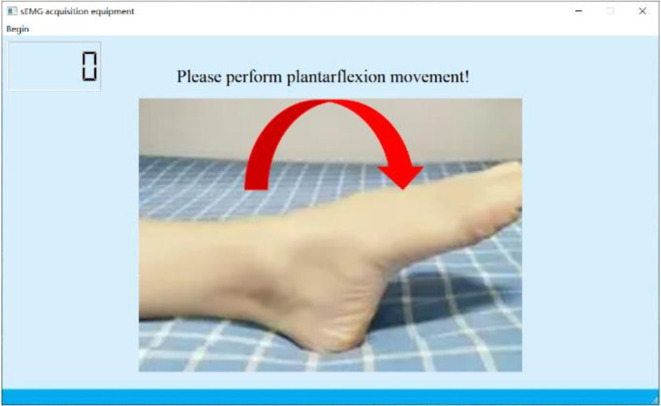
Screenshot of the guidance interface of the experiment.

During the experiment, the subjects were asked to place their “unaffected side foot” (left foot) on the moving platform. The experimental procedure is shown in Figure 6. At the beginning of the experiment, the subjects were asked to relax and stare at a screen. A NeuSen WM (Neuracle Co., Ltd., China) was used as the sEMG acquisition equipment. The sampling frequency was 1,000 Hz, and the EMG electrodes were Ag/Cl electrodes. Before the experiment, the skin surface was wiped with alcohol to remove stains from the skin surface so as to reduce the impedance between the electrodes and the skin. After the experiments began, the subjects were asked to perform the movements to the maximum range shown on the screen. The specific process was as follows: (a) remaining relaxed for 4 s; (b) performing the ankle joint motion shown on the screen with a rotational speed as constant as possible until the rotation limit was reached, and this process should be finished within 1 s; (c) maintaining the rotational angle for 3 s; and (d) resting for 4 s and starting the next motion. A set of experiments contained six ankle movements. The subjects had a 5-min break between each two sets of experiments to prevent muscle fatigue. The screenshot of the guidance interface of the experiment is shown in Figure 7. Each subject was asked to repeat the experiment 12 times under each load. In total, 216 (3 loads × 6 movements × 12 times) trials of experiments were completed by each subject. As a result, 6 × 216 = 1,296 trials were completed. All participants were trained to do the isokinetic contraction by watching an animation of a 1-s constant rotation of the ankle joint on the computer monitor before the experiment to improve the consistency of the ankle rotation speed during the experiment. At the same time, the subjects moved their ankles along with the animation. During the experiment, the same animation also appeared on the computer monitor to help the subjects move at a constant speed.

**Figure 8:**
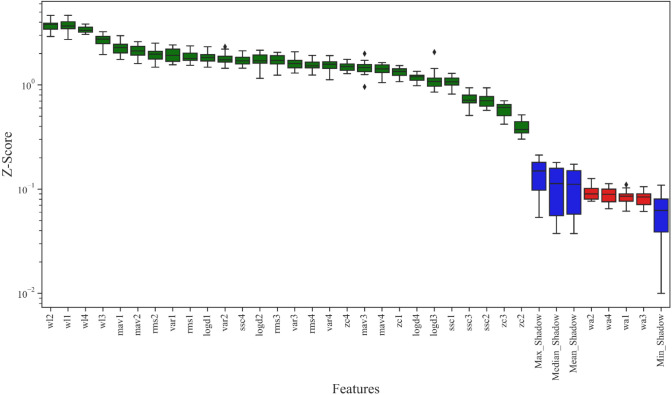
Result of the Boruta algorithm: *Z-Score* was used to represent the feature importance. The features suggested to be excluded are marked in red, features more related to the dependent variables are marked in green, and the shadow features are marked in blue.

The result of the Boruta algorithm is shown in Figure 8. The feature marked in blue is the shadow feature represented by Max_Shadow, Median_Shadow, Mean_Shadow, and Min_Shadow. The *Z-Score* of Max_Shadow was recorded as *Z*_max_, which was the criterion for judging the importance of features. Based on the *Z-Score* results, the feature suggested to be excluded was marked in red and seven features more related to ankle joint movements were marked in green. Therefore, seven features, including RMS, MAV, WL, ZC, SSC, VAR, and LogD, were selected to form the feature vector. In contrast, WA was not correlated with ankle joint movements and was excluded. Therefore, the number of selected features using the Boruta algorithm was 7.

Therefore, according to the division of the window, a small window of 20 ms could achieve a feature vector with a size of 7 × 4. Also, a large window of 210 ms could obtain a feature matrix with a size of 20 × 7 × 4. Since the time step of LSTM was 2, the input size of the CNN-LSTM model was 10 × 7 × 4. We evaluated the performance of several models (CNN-LSTM, CNN, and LSTM) commonly used in sEMG recognition, with the raw sEMG and the features of sEMG as the inputs, to demonstrate the superiority of our model and the superiority of using the features of sEMG as the input (Atzori et al., 2016; Gautam et al., 2020; Ma et al., 2020).

### 3.2. Experimental results

#### 3.2.1. Effect of load variation on the accuracy of movement classification

**Figure 9:**
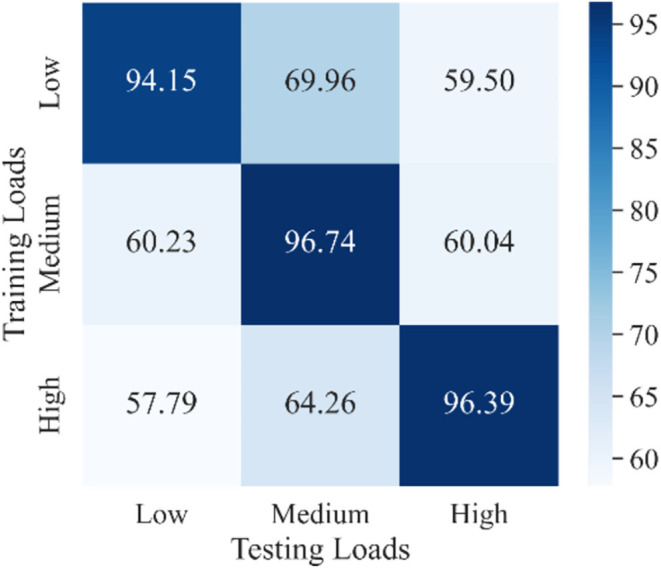
Confusion matrix of classification results for all subjects under every single load using a one-step method. Each element represents the accuracy of all subjects for corresponding training and testing loads. Darker color indicates higher accuracy. The main diagonal elements represent the accuracies where the load of training and testing sets are the same. The off-diagonal elements represent the accuracies where the load of training and the testing sets are different.

As mentioned in the Introduction section, the load variation has a great influence on the classification accuracy in motion recognition using sEMG signals (Al-Timemy et al., 2013; Tang et al., 2016). However, most recent studies did not take the load variation into account when they trained their movement classification model. In other words, their models were trained without distinguishing the load levels, which was referred in this study as a one-step method. The purpose of the experiment discussed in this section was to demonstrate the effect of load variation on the accuracy of movement classification and provide a comparison object for our proposed two-step method discussed in the next section. Three CNN-LSTM models with the same architecture were trained under each load and then tested using data from all three loads. The classification results for all subjects represented using a confusion matrix are shown in Figure 9. The horizontal coordinate of the confusion matrix represents the load of the testing set, and the vertical coordinate represents the load of the training set. Each element in the confusion matrix represents the accuracy (in percentage) of all subjects for corresponding training and testing loads. Among these, the main diagonal elements represent the accuracy values in percentage where loads of the training and testing sets are the same [intraload (Tang et al., 2016)], and the off-diagonal elements represent the accuracy values in percentage where loads of the training and testing sets are different [interload (Tang et al., 2016)]. For example, 94.15 shows that the accuracy of classifying ankle movements under the low load by the trained CNN-LSTM classifier under the low load was 94.15%. Further, 69.96 represents that the accuracy of classifying ankle movements under the medium load by the trained CNN-LSTM classifier under the low load was 69.96%. Also, 60.23 represents that the accuracy of classifying ankle movements under the low load by the trained CNN-LSTM classifier under the medium load was 60.23%. The average value and variance of accuracy under intraload, interload, and all loads are shown in Table 3. The overall accuracy in Table 3 represents the average accuracy of all subjects under intraload and interload when using the one-step method. The two-sample *t*-test was then conducted. Based on the test result (data were normal distribution using the Shapiro-Wilk test, *P* = 1.34E-18 < 0.001), it was suggested that a significant difference existed in the accuracy between intraload and interload. It indicated that the load variation had a substantial influence on the accuracy of movement classification. This revealed that the robustness of the one-step method was not good enough regarding the load variation.

**Table 3:** Average value and variance of accuracy under intraload, interload, and all loads using a one-step method.

	**Low**	**Medium**	**High**	**Average**
Intraload (mean ± sd)	94.15 ± 1.89	96.74 ± 2.05	96.39 ± 1.96	95.76 ± 1.96
Interload (mean ± sd)	59.01 ± 11.46	67.11 ± 7.32	59.77 ± 6.57	61.96 ± 8.45
Overall (mean ± sd)	70.72 ± 8.27	76.99 ± 5.56	71.98 ± 5.03	73.23 ± 6.29

#### 3.2.2. Comparison between the proposed two-step method and other conventional methods

In this study, we proposed a two-step method with a CNN-LSTM model to classify ankle movements. The first step was to calculate the RMS of sEMG and feed it into the Random Forest classifier for load classification. The Random Forest classifier was selected to recognize a specific load in this article because of its remarkable robustness and good resistance to overfitting (Zhou et al., 2019). Each subject performed six movements under a single load. Each movement was performed 12 times and lasted for 4 s. In order to reduce the influence of the movement process on the sEMG signals, 200 ms signals before and after each movement were removed. Therefore, each movement lasted for 3.6 s. The total length of sEMG signals recorded for each subject was 6 × 12 × 3.6 = 259.2 s. The number of samples per subject under a single load can be then calculated using equation (3).


(3) $$N_{s}=\frac{T-window\;length}{sliding\;length}$$

where *Ns* is the number of samples per subject under a single load, and *T* is the effective time of sEMG signals from each subject under a single load. Since the sliding length was 120 ms and the window length was 210 ms, the number of samples per subject under a single load was calculated to be 2,158.

Therefore, the number of samples per subject under all loads was 6,474. Since these samples were randomly divided into a training set and a testing set using a ratio of 8:2, the number of training and testing samples per subject under all loads was 5,179 and 1,295, respectively. Therefore, the number of training and testing samples for six subjects under three loads was 5,179 × 6, and 1,295 × 6, respectively. The total number of samples was 38,844, comprising six subjects under three load levels. In the training phase, a five-fold cross-validation strategy (where four-folds were used for training and one-fold was used for validation) was used to improve the consistency of the classification results. As a result, the number of samples for the training set under the low load recognized by the Random Forest classifier was 10,325 and the number of samples for the testing set was 2,623. The number of samples for the training set under the medium load recognized by the Random Forest classifier was 10,375, and the number of samples for the testing set was 2,573. The number of samples for the training set under the high load recognized by the Random Forest classifier was 10,374, and the number of samples for the testing set was 2,574. The training and testing sets were not of exactly the same size under each load level because of the randomness when dividing the datasets.

The classification results of the Random Forest classifier were then obtained (Table 4). The data in Table 4 represent the number of samples predicted to the corresponding load of the column. The average accuracy of the Random Forest classifier for all three loads was 92.81%, which could fulfill our requirements. In the second step, according to the identified load category, the features of sEMG were sent to the trained CNN-LSTM model with the same load category for movement classification.

**Table 4:** Results of load classification.

		**Predicted Load**			
		**Low**	**Medium**	**High**	**Acc**
Real Load	Low	2,430	150	43	92.64%
	Medium	61	2,407	105	93.55%
	High	62	138	2,374	92.23%
	Overall	2,553	2,695	2,522	92.81%

The results of movement classification are shown in Table 5. The first three columns of data in Table 5 show the number of samples in the classified load category that the movements are correctly identified, and the last column is the accuracy of movement classification. Using Table 3 as a reference, we could clearly see the advantages of the two-step method. The accuracy under low, medium, and high loads increased from 70.72%, 76.99%, and 71.98% to 91.42%, 95.22%, and 93.90%, respectively. The overall accuracy of movement classification increased from 73.23% to 93.50%. In addition, a two-sample *t*-test was conducted. The *P*-value of overall accuracy between the two-step and conventional methods was 7.90E-4. It was suggested that a significant difference existed in the accuracy between these two methods. Therefore, it was concluded that the proposed two-step method could greatly improve the accuracy of classification compared with the conventional one-step method.

**Table 5:** Results of movement classification using our proposed two-step method.

		**Predicted Load**			
		**Low**	**Medium**	**High**	**Acc**
Real Load	Low	2,284	92	22	91.42%
	Medium	45	2,330	75	95.22%
	High	42	84	2,291	93.90%
	Overall	2,371	2,506	2,388	93.50%

#### 3.2.3. Comparison among different models

**Figure 10:**
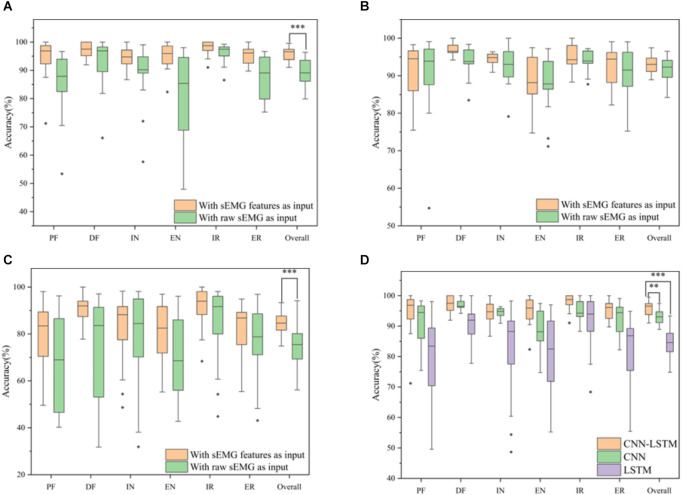
Classification accuracy for six movements using different models. **(A)** CNN-LSTM model with sEMG features and raw sEMG as the inputs. **(B)** CNN model with sEMG features and raw sEMG as the inputs. **(C)** LSTM model with sEMG features and raw sEMG as the inputs. **(D)** Classification accuracy for six movements using different network architectures with feature extraction (*P-value < 0.05, **P-value < 0.01, and ***P-value < 0.001).

Several model architectures with the sEMG features and the raw sEMG as inputs were compared to verify the performance of the proposed CNN-LSTM model with sEMG features as the input, as shown in Figure 10. It was obvious that the accuracy with sEMG features as the input was higher than that with raw sEMG as the input when using the CNN-LSTM, CNN, and LSTM models. The accuracy of the CNN-LSTM, CNN, and LSTM models with sEMG features as the input was 6.85%, 1.33%, and 9.00% higher than that of the corresponding models with raw sEMG as the input, respectively. A statistical analysis was conducted to explore the influence of different inputs on accuracy. According to the two-sample *t*-test (data were normal distribution using the Shapiro-Wilk test), the *P*-value of the CNN-LSTM, CNN, and LSTM models was 1.16E-05, 0.17, and 8.50E-04, respectively. It was suggested that a significant difference existed in the accuracy between sEMG features and raw sEMG as the inputs when using the CNN-LSTM and LSTM models. Meanwhile, we removed the Boruta algorithm and tested the CNN–LSTM model using all eight time-domain features to verify the significant improvement using the Boruta algorithm. The results showed that the classification accuracy of the CNN-LSTM model decreased from 95.73% to 92.84% after the Boruta algorithm was removed. A two-sample *t*-test (*P* = 0.004 < 0.01) was conducted, which showed that the performance of the model significantly improved when using the Boruta algorithm. In addition, the parameters of the CNN-LSTM model with sEMG features as the input was 155,042, while the parameters of the CNN-LSTM model with raw sEMG as the input was 331,938. Therefore, the computation cost of our method was only 46.70% of that of the conventional method. In addition, we calculated the time required to train these three models with sEMG features and with sEMG as the inputs. The total time required for each model with sEMG features as the input was equal to the time required for feature extraction plus the time required for training. The total time required by the CNN-LSTM, CNN, and LSTM models with sEMG features as the input was 34.48 s, 19.95 s, and 20.63 s, which was 44.92 s, 27.54 s, and 82.192 s less than that of the corresponding models with raw sEMG as the input, respectively. Moreover, the identification time of a single sample was calculated. The average identification time of a single sample required by the CNN-LSTM, CNN, and LSTM models with sEMG features as the input was 8.03 ms, 3.09 ms, and 3.20 ms, which was 16.57 ms, 11.62 ms, and 28.66 ms less than that of the corresponding models with raw sEMG as the input, respectively, guaranteeing the real-time control of rehabilitation robots.

Our model was compared with CNN, LSTM, SVM models used in the study by Hooda and Kumar (2021), the CNN-LSTM model used in the study by Cheng et al. (2020), and the CMNN model used in the study by Chen et al. (2019) to verify the superior performance of the proposed model. Figure 10D, Tables s 6 and 7 show the results of the aforementioned evaluation. It is worth mentioning that the results of the CNN model, LSTM model, and our proposed model were from the classification performance with the same dataset collected in the present study, whereas the results of the SVM (Hooda and Kumar, 2021), CNN-LSTM (Cheng et al., 2020), and CMNN (Chen et al., 2019) models were from the reported conclusions in the existing studies (Chen et al., 2019; Cheng et al., 2020; Hooda and Kumar, 2021). The accuracy of the CNN-LSTM model was 2.64% and 11.19% higher than that of the CNN and LSTM models, respectively. A two-sample *t*-test was conducted. According to the test result (data were normal distribution using the Shapiro-Wilk test), the *P*-value between the CNN-LSTM and CNN models was 0.002 (less than 0.01), and the *P*-value between the CNN-LSTM and LSTM models was 5.07E-10 (less than 0.001). It was suggested that a significant difference existed in the accuracy between the CNN-LSTM, CNN, and LSTM models. It indicated that the CNN-LSTM model had significant superiority over the CNN and LSTM models. Compared with the SVM classifier proposed by Hooda and Kumar (2021) the accuracy of the CNN-LSTM model was 3.13% higher. Moreover, the CNN-LSTM model proposed in this article could classify six movements including PF, DF, IV, EV, IR, and ER, whereas the SVM classifier proposed by Hooda and Kumar (2021) could only classify four movements, namely PF, DF, IV, and EV. Compared with the CMNN classifier proposed by Chen et al. (2019) and the CNN-LSTM hybrid model proposed by Cheng et al. (2020), the accuracy of the CNN-LSTM model was 1.17% and 1.82% lower, respectively. However, the CMNN model can only classify two movements, including IV and EV, and the model proposed by Cheng et al. (2020) can only classify four movements, including PF, DF, IV, and EV. In addition, the raw sEMG instead of features was fed into the model proposed by Cheng et al. (2020), which involved huge computational costs.

**Table 6:** Performance of different network architecture and inputs.

**Model**	**Inputs**	**Total Accuracy (%)**	**Training Time (s)**	**Identification Time of a Single Sample (ms)**
CNN-LSTM	features	95.73 ± 3.92	34.48	8.03
CNN-LSTM	raw sEMG signals	88.88 ± 9.13	79.40	24.60
CNN	features	93.09 ± 4.01	19.95	3.09
CNN	raw sEMG signals	91.76 ± 8.02	47.49	14.71
LSTM	features	84.54 ± 10.22	20.63	3.20
LSTM	raw sEMG signals	75.54 ± 17.88	102.82	31.86

**Table 7:** Comparison with the state-of-the-art method.

**Model**	**Category**	**Total Accuracy (%)**
SVM (Hooda and Kumar, 2021)	4 movements (PF, DF, IV and EV)	92.60 ± 6.91
CMNN (Chen et al., 2019)	2 movements (IV and EV)	96.90
CNN-LSTM (Cheng et al., 2020)	4 movements (PF, DF, IV and EV)	97.55 ± 1.93
Our model	6 movements (PF, DF, IV, EV, IR, and ER)	95.73 ± 3.92

Please note that the differences between the proposed CNN-LSTM and the CNN-LSTM proposed by Cheng et al. (2020) are as follows. (1) The input processes for those two models were different. In our proposed CNN-LSTM, the sEMG signals collected from four channels were fed into CNN respectively. They were flattened and concatenated after convolution. In the CNN-LSTM proposed by Cheng et al. (2020), the sEMG signals of all channels were fed together for convolution. (2) The outputs of these two modes were different. The output of our model was six categories, while that of the CNN-LSTM proposed by Chen et al. (2020) was four categories; and (3) The convolution layer, LSTM layer, and Dense layer of these two models had different number of layers and parameters.

## 4. Discussion

### 4.1. Classified ankle movements

As mentioned earlier, the current literature mainly focused on gait recognition rather than on ankle movement classification for lower-limb rehabilitation training (Varol et al., 2010; Joshi et al., 2013; Gautam et al., 2020). The reason might be that the ankle joint could perform six movements, and it is more difficult to accurately decode those six movements than gait recognition using sEMG signals. Two ankle movements including IV and EV could be classified with an accuracy of 96.90% by the method proposed by Chen et al. (2019). The models proposed in the studies by Hooda and Kumar (2021) and Cheng et al. (2020) could classify four ankle movements (PF, DF, IV, and EV) with an accuracy of 92.60% and 97.55%, respectively. IR and ER movements could be recognized incorrectly because the PF/DF of the ankle was always accompanied by IR/ER (Chen, 2018). Whether applying the methods used in these previous studies could classify these six movements and maintain high accuracy remains to be explored.

In this study, our model can classify six ankle movements with an accuracy of 95.70%. Compared with the models in the aforementioned studies, our model can classify more ankle movements with relatively good accuracy. Our target is to use the decoded movements of the unaffected side to control the robot-assisted motion on the affected side of stroke patients.

### 4.2. Feature extraction

The timing of paired human movement intent and associated feedback is critical to induce neuroplasticity (estimated to be within 300 ms) during rehabilitation training (Hudgins et al., 1993). Therefore, reducing decoding time movement intent from sEMG is an important task. Deep learning models such as CNN can be used as classifiers to classify ankle movements. However, normally raw sEMG signals are directly fed into the model, which brings heavy calculation burden; thus, the real-time performance cannot be guaranteed. In our study, seven time-domain features were selected using the Boruta algorithm. Then, these features were fed into our CNN-LSTM model. To our best knowledge, this is the first time that the Boruta algorithm is used for feature selection of sEMG, and it is also the first time that selected features rather than raw sEMG are fed into a CNN-LSTM model. In addition, adding more layers to the network might improve the accuracy. However, the computational cost will increase, which will affect the real-time performance. The aim of selecting time-domain features with the Boruta algorithm and feeding them into the network is to find a balance, which can not only obtain high accuracy but also meet the requirements of real-time performance.

**Figure 11:**
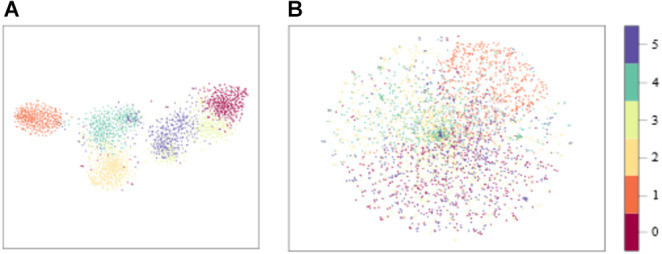
Distribution of features. **(A)** CNN-LSTM model input with sEMG features as the input. **(B)** CNN-LSTM model input with raw sEMG as the input.

The distribution of model input with sEMG features and raw sEMG is visualized in Figure 11 with *t*-Distributed Stochastic Neighbor Embedding (*t*-SNE), where the dimension of model input was reduced from high dimension to 2D. We could see that the boundaries of each class with sEMG features as the input were clearer than those with raw sEMG as the input. As a result, the convergence speed of the model with sEMG features as the input would be larger. The time required to train these three models with sEMG features and raw sEMG as the inputs was calculated. The total time required by these three models with sEMG features as the input is all less than that of the corresponding models with raw sEMG as the input. Also, the time required by the CNN-LSTM model was longer than that required by the CNN and LSTM models. This might be because the CNN-LSTM model is more complex and has more parameters. In the future study, we should find ways to reduce the parameters of the CNN-LSTM model and the training time while maintaining high accuracy. On the contrary, using our proposed CNN-LSTM model, the ankle movement classification time of a single sample was only 8.03 ms, which was about 5 ms longer than that using the CNN or LSTM model. It would not be difficult to meet the real-time requirement (300 ms) of rehabilitation even considering other time delay factors in the rehabilitation system (Hudgins et al., 1993). As shown in Table 6; the average accuracy of the CNN-LSTM, CNN, and LSTM models with features of sEMG as the inputs was higher than that with the raw sEMG signals as the input. In addition, the statistical analysis results showed that the input of the CNN-LSTM and LSTM models had a significant impact on the accuracy whereas no significant difference on accuracy was found between the CNN-LSTM and CNN models. This might be because the CNN model has a good deep feature extraction performance. In the future study, the specific reason should be found.

### 4.3. Load variation

Different load levels will lead to different levels of muscle activity, and sEMG reflects the level of muscle activity (Kiguchi and Hayashi, 2012). If the classifier trained under a single load is used to classify the sEMG signals under other loads, the accuracy will be greatly reduced (33.80%), as shown in Table 3. Tang et al. (2016) found that putting EMG signals under all loads together into the model could not achieve an ideal performance when decoding the elbow joint angle. In this study, we used a two-step method to reduce the effect of load variation on the classification results of ankle movements. In the proposed two-step method, multiple CNN-LSTM models were trained under different loads. In this study, we trained three CNN-LSTM models under three loads (low, medium, and high). When adopting the two-step method, a random forest classifier was used to classify the load first, and then the corresponding CNN-LSTM model was selected to classify the movement. As shown in Tables s 3 and 5, the overall accuracy improved from 73.23% to 93.50% using the two-step method. Unfortunately, errors existed (7.19%) when using the Random Forest classifier to classify the load. In other words, some signals of a specific load were fed into trained models under a different load level which it should not belong to. In future studies, improving the accuracy of load level recognition is the direction of our efforts. In addition, we only used three load levels (low, medium, and high) in this study. However, in practical applications, the load may not be within the training range. Expanding the training pool and dividing the load levels more finely may be a solution. Another factor that has a certain impact on accuracy is fatigue (Enoka et al., 2011). In this study, the problem was addressed by giving the subjects plenty of time to rest during the experiment. Our data were not collected under fatigue. In future studies, fatigue should also be taken into account.

### 4.4. Different models

In this study, we adopted the CNN-LSTM model to classify ankle movements. The model consisted of two convolution layers, one LSTM layer, and two dense layers. The hyperparameters of our model were mainly determined with reference to previous studies and then manually tuned *via* experience (Gautam et al., 2020). We compared the performance of our model with that of other models and performed the statistical analysis. As shown in Table 6, the accuracy of our CNN-LSTM model was higher than that of the CNN and LSTM models. The statistical analysis results indicated that the CNN-LSTM model had significant superiority over CNN and LSTM models. Furthermore, the validation of our model for ankle movements classification is shown in Table 7 along with a comparison with the CMNN (Chen et al., 2019), CNN-LSTM (Cheng et al., 2020), and SVM (Hooda and Kumar, 2021) state-of-the-art models. Unfortunately, since not many researchers focused on classifying the ankle movements alone using sEMG signals, finding any suitable public datasets to further verify the robustness of our method was difficult. Please note that, since detailed information about the computational costs of these three models [SVM (Hooda and Kumar, 2021), CNN-LSTM (Cheng et al., 2020), and CMNN (Chen et al., 2019) models] was not found, only the classification accuracy was compared between these three models and our proposed CNN-LSTM model. Further investigation regarding the computation complexity or computation efficiency of those models are desired in the future study.

As mentioned in Section “2.4 Network architecture”, after the pre-processing, a feature matrix with the size of 20 × N was obtained for each channel (four channels in total). The feature matrix used in our proposed CNN–LSTM model, the conventional CNN, and LSTM models was the same. However, the input dimensions to these models were different because the requirements of these three models were different. The input dimension of CNN was 20 × N. The input of the traditional LSTM model is required to be a one-dimensional vector. Therefore, in this study, the feature matrix was flattened to become a one-dimensional input vector before being fed into the LSTM model. The input dimension for the proposed CNN-LSTM model is related to the time step. Because the time step was set to 2, the 20 × N feature matrix was divided into two 10 × N input matrices before being fed into the CNN-LSTM model. Each 10 × N matrix was used as the input for each step. Although the dimensions were different, the feature matrix used in these three models was the same. Therefore, in this study, we considered that the fairness of comparison could be guaranteed. In future studies, the influence of the input dimensions should be investigated in detail. Another possible direction is to find a way to keep the input dimension the same when using different models.

### 4.5. Limitations and future directions

This study had a few limitations. First, in our study, all the subjects were healthy male subjects and the number of subjects was not large enough. The age of the subjects was 22–25 years. In future studies, the diversity of the subjects should be increased. Moreover, the sEMG signals on the unaffected side of stroke patients may be different from those of healthy people. Hence, in future studies, the validation experiments should involve stroke patients.

The second limitation was the classification speed. Although we adopted some methods such as feature extraction to improve the classification speed, using the CNN-LSTM model still required more parameters. Most of the parameters were in the LSTM layer. However, the LSTM layer was critical in our model, which was used to extract the correlation on the time sequence. Therefore, in future studies, we should try to reduce the parameters of the LSTM layer while ensuring accuracy.

In this study, we proposed a CNN-LSTM model for the first time to classify ankle movement using sEMG signals. Compared with the previous methods (Varol et al., 2010; Joshi et al., 2013; Gautam et al., 2020), the performance of our method has been already greatly improved. As we all know, deep learning develops rapidly. In the last few years, some novel models have been developed and used to classify the movements of the hand and wrist joints, for example, CNN-BiLSTM (Nguyen-Trong et al., 2021; Tripathi et al., 2022) and Graph Convolutional Network (GCN; Lai et al., 2021; Yang et al., 2022). However, to the best of our knowledge, they have not been used in studies to classify ankle movements. In future studies, we will adopt these novel models and compare them with our proposed ones.

The method we proposed in this study could accurately classify these six ankle movements using sEMG signals. As we all know, the movement of the human wrist is quite similar to that of the ankle joint. Therefore, we can try to apply our method to the decoding of human wrist movements in the future.

In this study, we provided the basis of the ankle movement classification for robot-assisted bilateral rehabilitation training. Although some researchers have realized robot-assisted bilateral rehabilitation training for patients based on sEMG signals for other parts of the body, for example, the hands (Leonardis et al., 2015), this concept has not been applied to ankle rehabilitation. Realizing the concept of using the unaffected side for the robot-assisted motion control on the affected side in ankle rehabilitation may be challenging due to the different motion patterns or constraints between the intact side and the affected side for stroke survivors. These should be explored in future studies.

## 5. Conclusion

In this article, a time-domain feature selection method of the sEMG, a CNN-LSTM model, and a two-step method were proposed to decode the movement intention of the ankle joint for bilateral ankle rehabilitation training to classify more ankle movements, reduce the computational cost, and minimize the influence of load variation on classification results. For the first time, the Boruta algorithm was used in this study to select time-domain features of sEMG. The selected features rather than raw sEMG were fed into the CNN-LSTM model. Hence, the parameters of the model were reduced from 331,938–155,042. Experiments were conducted to verify the proposed method. The results showed that our method could classify six ankle movements with relatively good accuracy (95.70%). The total time required by the CNN-LSTM, CNN, and LSTM models with sEMG features as the input was all less than that of the corresponding models with raw sEMG as the input. In addition, the accuracy of the CNN-LSTM, CNN, and LSTM models with sEMG features as the input was all higher than that of the corresponding models with raw sEMG as the input. The overall accuracy was improved from 73.23% to 93.50% using our two-step method for classifying the ankle movements with different loads. Our proposed CNN-LSTM model had the highest accuracy in ankle movements classification compared with the CNN, LSTM, and SVM models.

## Data availability statement

The datasets presented in this study can be found in online repositories. The names of the repository/repositories and accession number(s) can be found below: https://ieee-dataport.org/documents/semg-signals-ankle-movement-under-different-loads.

## Ethics statement

The studies involving human participants were reviewed and approved by Institutional Review Board of Xi’an Jiaotong University. The patients/participants provided their written informed consent to participate in this study.

## Author contributions

ML and JW: conceptualization and methodology, experiment design, data analysis. ML: supervision. JW and SY: investigation. ML, JW, JX, GX, and SL: writing. ML and SL: funding acquisition. All authors contributed to the article and approved the submitted version.
